# Peripheral blood mononuclear cell low molecular mass protein 7 in acute ischemic stroke: vertical change from admission to discharge and correlation with disability, stroke recurrence, and death

**DOI:** 10.3389/fimmu.2024.1296835

**Published:** 2024-02-08

**Authors:** Lujia Hou, Yanlei Zhang

**Affiliations:** ^1^ Department of Neurology, YongJia People’s Hospital, Wenzhou, China; ^2^ Department of Neurology, The First Affiliated Hospital of Wenzhou Medical University, Wenzhou, China

**Keywords:** acute ischemic stroke, low molecular mass protein 7, T cell subsets, disease severity, prognosis

## Abstract

**Objective:**

Low molecular mass protein 7 (LMP7) aggravates abnormal T cell differentiation and atherosclerosis, but its clinical role in acute ischemic stroke (AIS) is still unclear. This study aimed to investigate the correlation of peripheral blood mononuclear cell (PBMC) LMP7 with T cell subsets, disease severity, and prognosis in AIS patients.

**Methods:**

A total of 162 AIS patients were enrolled for detecting PBMC LMP7 and T helper (Th) 1, Th2, and Th17 cells via reverse transcriptase-polymerase chain reaction and flow cytometry, respectively. In addition, PBMC LMP7 at discharge was also quantified.

**Results:**

Increased LMP7 at admission was associated with decreased Th2 cells (*P*=0.014), elevated Th17 cells (*P*<0.001), C-reactive protein (*P*=0.005), National Institutes of Health Stroke Scale (NIHSS) score (*P*=0.007), and disease severity (defined by NIHSS score) (*P*=0.010). LMP7 at admission reflected a high risk of stroke recurrence (area under curve (AUC): 0.748, 95% confidence interval (CI): 0.564-0.932), but not mRS score at month 3 (M3) >2 (AUC: 0.585, 95%CI: 0.479-0.691), or death (AUC: 0.723, 95%CI: 0.338-1.000). LMP7 at discharge was reduced compared to that at admission (*P*<0.001). LMP7 at discharge was positively correlated with the risk of stroke recurrence (AUC: 0.849, 95%CI: 0.735-0.963) and death (AUC: 0.919, 95%CI: 0.836-1.000), but had a weak capacity to reflect mRS score at M3 >2 (AUC: 0.671, 95%CI: 0.578-0.765).

**Conclusion:**

PBMC LMP7 positively correlates with Th17 cells, inflammation, and disease severity in AIS patients, meanwhile, its level at discharge shows a good ability to reflect the risks of stroke recurrence and death.

## 1 Introduction

Acute ischemic stroke (AIS), a leading cause of disability, affects approximately 0.7 million people each year in the United States and 2 million people each year in China (1, 2). AIS is an acute arterial condition that results in cerebral ischemia due to insufficient blood flow to the brain (1, 3). The Trial Org 10172 in Acute Stroke Treatment (TOAST) classification subdivides AIS into cardioembolism, small-vessel occlusion, large-artery atherosclerosis (the most common), and stroke of undetermined etiology (3–5). Intravenous thrombolysis within 4.5 hours after onset, mechanical thrombectomy within 24 hours after onset, and dual antiplatelet therapy are common treatments for AIS patients (6). AIS patients commonly have a high risk of disability, recurrence, and even repeated recurrence as well as death (7–9). Thus, finding biomarkers for monitoring disease severity and estimating the risk of disability, recurrence, and death of AIS patients is a worthwhile endeavor.

Low molecular mass protein 7 (LMP7), a key component of the immunoproteasome, is reported to be involved in T cell differentiation, inflammatory response, and lipid metabolism (10–13). For instance, a study shows that LMP7 deficiency suppresses the generation of T helper 1 (Th1) and Th17 cells by reducing signal transducers and activators of transcription (STAT) 1 and blocking phosphorylation of STAT3 *in vivo* (11). One study has indicated that LMP7 inhibition has an anti-inflammatory effect by limiting interleukin (IL)-6 secretion (12). In addition, a previous investigation has illustrated that LMP7 deficiency reduces lipid absorption and inhibits the increase of plasma triglyceride levels in oral oil administration or mice fed a high-fat-diet (13). The dysregulation of T cell subsets, inflammation, and lipid metabolism mentioned above have important regulatory effects on atherosclerosis and brain injury (14–17). Therefore, LMP7 may have an indirect regulatory effect on atherosclerosis and brain injury.

Moreover, several studies also indicate that LMP7 possesses a direct regulatory effect on atherosclerosis and brain injury (10, 18). One study indicates that LMP7 plays a pro-atherogenic role by inhibiting mer tyrosine kinase (MERTK)-mediated efferocytosis and enhancing atherosclerotic plaque instability in apolipoprotein E knockout mice (18). Furthermore, another study reveals that LMP7 aggravates brain injury by triggering inflammatory responses via phosphatidylinositol 3-kinase/protein kinase B (PI3K/Akt) signaling in hypoxic-ischemic brain damage rats (10). Though these studies show the engagement of LMP7 in atherosclerosis and ischemic brain injury (10, 19, 20), the clinical role of LMP7 in AIS patients is unclear so far.

Hence, this study detected peripheral blood mononuclear cells (PBMC) LMP7 at admission and discharge, aiming to investigate its correlation with T cell subsets, disease severity, and prognosis in AIS patients.

## 2 Methods

### 2.1 Patients

A total of 162 AIS patients who visited our hospital between August 2021 and December 2022 were enrolled in this research. The inclusion criteria were: 1) diagnosed as AIS by Chinese guidelines for diagnosis and treatment of AIS 2018 (21); 2) aged ≥18 years old; 3) had the ability to cooperate with relevant assessment; 4) willing to cooperate with peripheral blood collection. The exclusion criteria were: 1) intracerebral hemorrhage or transient ischemic attack; 2) global or receptive aphasia, hearing impairment, or physical disability previously; 3) expected survival <3 months; 4) women who were pregnant or lactating. This research acquired approval from the Ethics Committee of the First Affiliated Hospital of Wenzhou Medical University. Written informed consent was gathered from patients or their families.

### 2.2 Characteristics collection

Characteristics were collected from AIS patients at admission, which contained age, gender, body mass indexes (BMI), smoke, hypertension, hyperlipidemia, diabetes mellitus, cardiovascular disease, time since symptom to admission, National Institutes of Health Stroke Scale (NIHSS) score, treatment, white blood cell (WBC), fasting plasma glucose (FBG), serum creatinine (Scr), triglyceride (TG), total cholesterol (TC), low-density lipoprotein cholesterol (LDL-C), high-density lipoprotein cholesterol (HDL-C), prothrombin time (PT), activated partial thromboplastin time (APTT), fibrinogen (FIB), D-dimer, and C-reactive protein (CRP).

### 2.3 Peripheral blood collection and detection

Peripheral blood was gathered from AIS patients at admission and separated into two parts immediately. One part was used to determine the proportion of Th1, Th2, and Th17 cells in CD4^+^ T cells directly; the other part was used to isolate PBMC for LMP7 detection. At discharge, the peripheral blood was gathered again for PBMC LMP7 detection as well.

For Th1, Th2, and Th17 cell proportions in the CD4^+^ T cells detection. The CD4^+^ T cells were first isolated (Human CD4^+^ T Cell Isolation Kit, No. Cat. MAGH102, Bio-Techne, China). Then those proportions were determined by flow cytometric (FCM) analysis (FlowX Human Th1, Th2, and Th17 Cell Multi-Color Flow Cytometry Kits, No. Cat. FMC009B, FMC011B, and FMC007B, Bio-Techne, China). After that, a FACS Canto II flow cytometer (BD, USA) was utilized to read data. All procedures were conducted based on the kit’s instructions.

PBMC LMP7 detection was conducted by reverse transcriptase-polymerase chain reaction (RT-qPCR) assay. Total RNA was extracted for cDNA synthesis, then followed by an RT-qPCR analysis. The kits were as follows: TRIzol™ Reagent, No. Cat. 15596018, Invitrogen™, USA; iScript™ Reverse Transcription Supermix, No. Cat. HY-000338, Bio-Rad, USA; and KOD SYBR^®^ qPCR Mix, No. Cat. QKD-201, Toyobo, Japan. The primers were as follows: 1) LMP7, forward: 5’-CGCTCTTGTGGGTGACTACA-3’, reverse: 5’-TGGGTAGGGTCGTGTCATCT-3’; 2) GAPDH, forward: 5’-GAGTCCACTGGCGTCTTCAC-3’, reverse: 5’-ATCTTGAGGCTGTTGTCATACTTCT-3’. GAPDH was set as an internal reference and the median △Ct of PBMC LMP7 at admission was set as a reference for calculating △△Ct. The PBMC LMP7 was calculated via the 2^-△△Ct^ method (10).

### 2.4 NIHSS and modified Rankin Scale scores

NIHSS score was performed to measure stroke severity at admission, which was scaled from 0 to 42. A higher NIHSS score represented a more severe stroke, which was classified into mild (0~4), moderate (5~15), moderate to severe (16~20), and severe (>20) (22). The mRS score was used for assessing the level of disability at 3 months after admission (M3), which was scaled from 0 to 6. A higher mRS score represented a worse functional dependency, and 6 represented a death. In this research, the mRS score was divided into ≤2 and >2 levels for analyses (23).

### 2.5 Follow-up

Patients received routine follow-ups, and the median follow-up time was 9.3 months (range, 1.5~18.4 months). During the period, recurrence and death statuses were recorded. Nine patients (5.6%) presented with recurrence and three patients (1.9%) died.

### 2.6 Statistics

Data were imputed into SPSS v.26.0 (IBM, USA) for statistical analyses. The Spearman test was used for analyzing the correlation between variables. The Wilcoxon rank sum test, *χ^2^* test, and Wilcoxon signed-rank test were performed for comparison analyses. The receiver operating characteristic (ROC) curves were utilized to show the distinguished abilities of PBMC LMP7. Data of patients who had disease recurrence during hospitalization were not included in the analysis. Statistics was defined as *P*<0.050.

## 3 Results

### 3.1 Baseline clinical characteristics

The median (interquartile range (IQR)) age of AIS patients was 67.0 (60.0-75.0) years. There were 47 (29.0%) women and 115 (71.0%) men. The median (IQR) time since symptom to admission was 4.0 (3.0-7.0) h. The median (IQR) NIHSS score was 8.0 (5.0-12.0). The median (IQR) Th1, Th2, and Th17 cells were 16.5 (13.0-20.3) %, 11.2 (8.8-15.1) %, and 2.8 (1.6-4.9) %, respectively. The median PBMC LMP7 was 1.000 (0.786-1.610). The detailed clinical information is listed in **Table 1**.

**Table 1:** Characteristics at admission.

Characteristics	AIS patients (N = 162)
Age (years), median (IQR)	67.0 (60.0-75.0)
Gender, No. (%)	
Female	47 (29.0)
Male	115 (71.0)
BMI (kg/m^2^), mean ± SD	25.5 ± 2.7
Smoke, No. (%)	60 (37.0)
Hypertension, No. (%)	124 (76.5)
Hyperlipidemia, No. (%)	78 (48.1)
Diabetes mellitus, No. (%)	43 (26.5)
Cardiovascular disease, No. (%)	62 (38.3)
Time since symptom to admission (h), median (IQR)	4.0 (3.0-7.0)
NIHSS score, median (IQR)	8.0 (5.0-12.0)
Treatment, No. (%)	
MT	42 (25.9)
UK with IVT	65 (40.1)
rtPA with IVT	30 (18.5)
UK with IVT bridging to MT	19 (11.7)
rtPA with IVT bridging to MT	6 (3.7)
WBC (10^9^/L), median (IQR)	10.3 (7.7-13.0)
FBG (mmol/L), median (IQR)	5.5 (4.5-6.4)
Scr (μmol/L), mean ± SD	89.0 ± 19.4
TG (mmol/L), median (IQR)	1.8 (1.0-2.5)
TC (mmol/L), median (IQR)	4.5 (3.7-5.3)
LDL-C (mmol/L), median (IQR)	3.0 (2.3-3.9)
HDL-C (mmol/L), median (IQR)	1.0 (0.9-1.2)
PT (s), mean ± SD	11.7 ± 2.4
APTT (s), median (IQR)	28.0 (24.0-34.0)
FIB (g/L), mean ± SD	3.4 ± 0.7
D-dimer (mg/L), median (IQR)	0.2 (0.1-0.3)
CRP (mg/L), median (IQR)	4.8 (2.8-7.1)
Th1 cells (%), median (IQR)	16.5 (13.0-20.3)
Th2 cells (%), median (IQR)	11.2 (8.8-15.1)
Th17 cells (%), median (IQR)	2.8 (1.6-4.9)
PBMC LMP7, median (IQR)	1.000 (0.786-1.610)

AIS, acute ischemic stroke; IQR, interquartile range; BMI, body mass index; SD, standard deviation; NIHSS, national institutes of health stroke scale; MT, mechanical thrombectomy; UK, urokinase; IVT, intravenous thrombolysis; rtPA, recombinant tissue plasminogen activator; WBC, white blood cell; FBG, fasting plasma glucose; Scr, serum creatinine; TG, triglyceride; TC, total cholesterol; LDL-C, low-density lipoprotein cholesterol; HDL-C, high-density lipoprotein cholesterol; PT, prothrombin time; APTT, activated partial thromboplastin time; FIB, fibrinogen; CRP, C-reactive protein; Th1, T helper 1; Th2, T helper 2; Th17, T helper 17; PBMC, peripheral blood mononuclear cells; LMP7, low molecular mass protein-7. Kolmogorov–Smirnov test was used for the normality test. Continuous variables that did not obey a normal distribution were described by the median (IQR); while continuous variables that obeyed a normal distribution were described by mean ± SD.

### 3.2 Correlation of PBMC LMP7 at admission with T cell subsets, CRP, and NIHSS score

**Figure 1:**
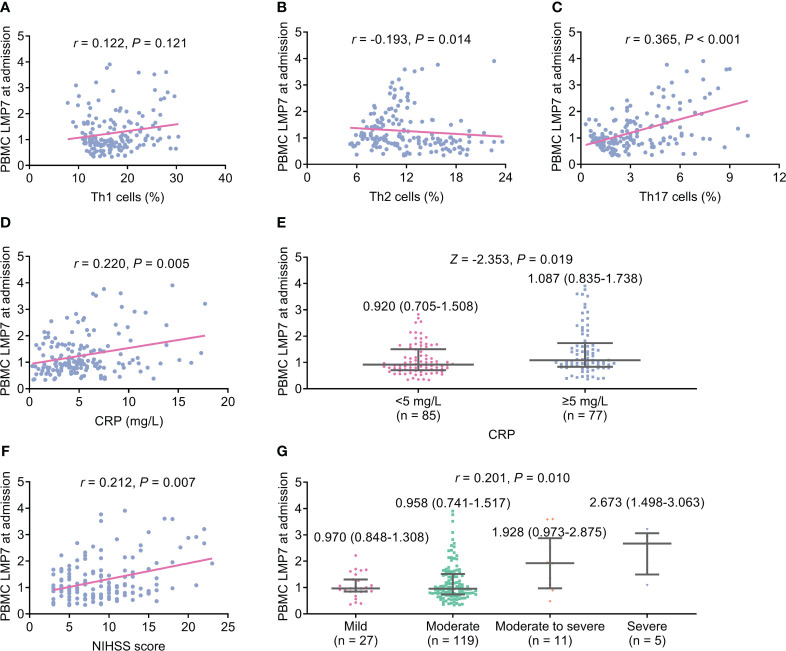
Elevated PBMC LMP7 at admission was correlated with decreased Th2 cells and increased Th17 cells, CRP, and NIHSS score in AIS patients. The correlation of PBMC LMP7 at admission with Th1 cells **(A)**, Th2 cells **(B)**, Th17 cells **(C)**, CRP **(D)**, CRP ≥5 mg/L **(E)**, NIHSS score **(F)**, and stroke severity classified by NIHSS score **(G)**.

PBMC LMP7 at admission was not correlated with Th1 cells (*P*=0.121, **Figure 1A**), but elevated PBMC LMP7 at admission was associated with decreased Th2 cells (*P*=0.014, **Figure 1B**) and increased Th17 cells (*P*<0.001, **Figure 1C**). PBMC LMP7 at admission was positively linked with CRP (*P*=0.005, **Figure 1D**). After dividing the patients into two categories by CRP level of 5 mg/L, PBMC LMP7 was positively linked with CRP ≥5 mg/L as well (*P*=0.019, **Figure 1E**). In addition, PBMC LMP7 at admission also exhibited a positive association with NIHSS score (*P*=0.007, **Figure 1F**) and stroke severity classified by NIHSS score (*P*=0.010, **Figure 1G**).

The non-linear analysis was performed, which showed that PBMC LMP7 at admission was not related to Th1 cells (*P*=0.106). While PBMC LMP7 at admission ≥1 was associated with reduced Th2 (*P*=0.012) but increased Th17 cells (*P*<0.001). In addition, PBMC LMP7 at admission ≥1 was linked with elevated CRP (*P*=0.045). However, PBMC LMP7 at admission was not varied between patients with CRP <5 mg/L and ≥5 mg/L (*P*=0.058). PBMC LMP7 at admission was not linked with NIHSS score (*P*=0.081), while PBMC LMP7 at admission ≥1 was related to enhanced stroke severity (*P*=0.047) (**Supplementary Table 1**).

### 3.3 Comparison of PBMC LMP7 at admission between patients with or without disability, recurrence, or death

**Figure 2:**
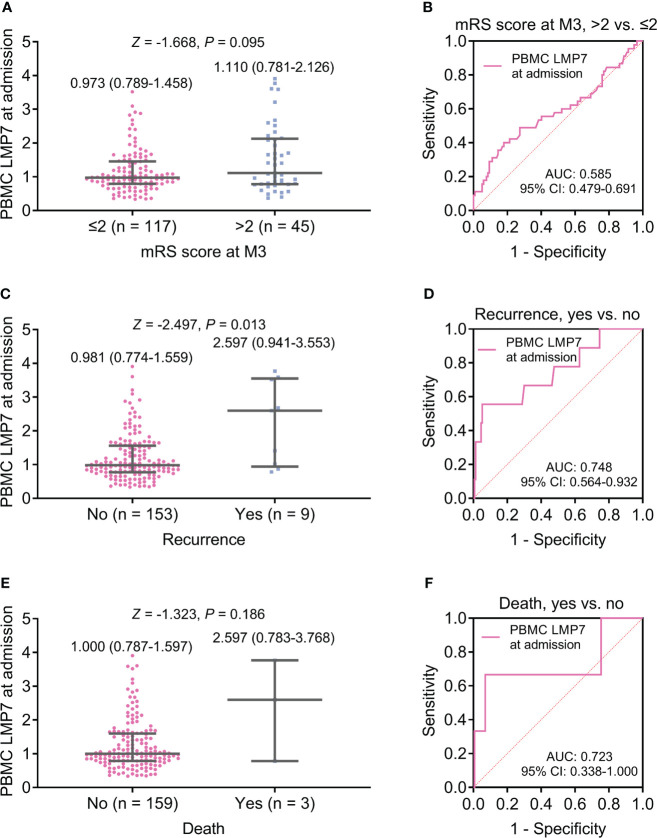
PBMC LMP7 at admission was elevated in AIS patients with recurrence compared to those without recurrence. Comparison of PBMC LMP7 at admission between patients with mRS score at M3 >2 and ≤2 **(A)**, and ROC analysis for its value on estimating the risk of mRS score at M3 >2 **(B)**. Comparison of PBMC LMP7 at admission between patients with and without recurrence **(C)**, and ROC analysis for its value on estimating recurrence risk **(D)**. Comparison of PBMC LMP7 at admission between patients with and without death **(E)**, and ROC analysis for its value on estimating death risk **(F)**.

No difference was found in PBMC LMP7 at admission between patients with mRS score at M3 ≤2 and >2 (*P*=0.095, **Figure 2A**), and PBMC LMP7 lacked the ability to estimate the risk of patients with mRS score at M3 >2 (area under curve (AUC): 0.585, 95% confidence interval (CI): 0.479-0.691, **Figure 2B**). PBMC LMP7 at admission was elevated in patients with recurrence (n=9) compared to those without (n=153) (*Z*=-2.497, *P*=0.013, **Figure 2C**), and it disclosed a certain value for estimating the risk of recurrence (AUC: 0.748, 95%CI: 0.564-0.932, **Figure 2D**). Moreover, PBMC LMP7 at admission was of no difference between patients with and without death (*P*=0.186, **Figure 2E**), and it did not exhibit a value to estimate the risk of death (AUC: 0.723, 95%CI: 0.338-1.000, **Figure 2F**).

### 3.4 PBMC LMP7 change

**Figure 3:**
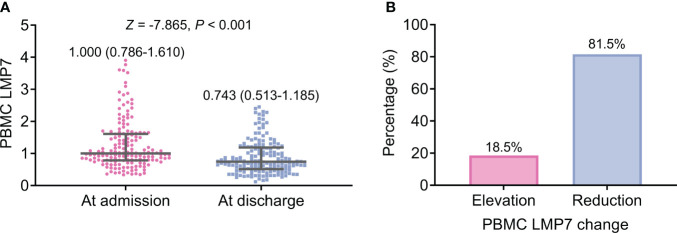
PBMC LMP7 at discharge was decreased than that at admission in AIS patients. Comparison between PBMC LMP7 at admission and at discharge in AIS patients **(A)**. The proportion of patients with elevated and reduced PBMC LMP7 from admission to discharge **(B)**.

PBMC LMP7 at discharge (median (IQR): 0.743 (0.513-1.185)) was reduced compared to PBMC LMP7 at admission (median (IQR): 1.000 (0.786-1.610)) (*P*<0.001, **Figure 3A**). Besides, PBMC LMP7 was increased from admission to discharge in 18.5% of patients, while 81.5% of patients had a reduced PBMC LMP7 from admission to discharge (**Figure 3B**).

### 3.5 Comparison of PBMC LMP7 at discharge between patients with or without disability, recurrence, or death

**Figure 4:**
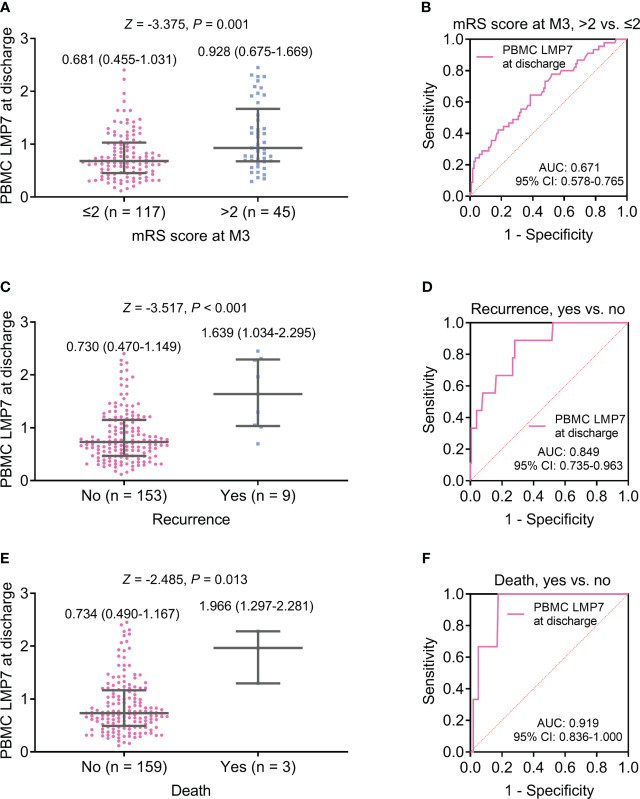
PBMC LMP7 at discharge was elevated in AIS patients with disability, recurrence, or death than those without. Comparison of PBMC LMP7 at discharge between patients with mRS score at M3 >2 and ≤2 **(A)**, and ROC analysis for its value on estimating the risk of mRS score at M3 >2 **(B)**. Comparison of PBMC LMP7 at discharge between patients with and without recurrence **(C)**, and ROC analysis for its value on estimating recurrence risk **(D)**. Comparison of PBMC LMP7 at discharge between patients with and without death **(E)**, and ROC analysis for its value on estimating death risk **(F)**.

PBMC LMP7 at discharge was elevated in patients with mRS score at M3 >2 compared to those with the score <2 (*P*=0.001, **Figure 4A**), but its ability to reflect the risk of mRS score at M3 >2 was not ideal (AUC: 0.671, 95%CI: 0.578-0.765, **Figure 4B**). PBMC LMP7 at discharge was increased in patients with recurrence (n=9) compared to those without (n=153) (*Z*=-3.517, *P*<0.001, **Figure 4C**), and it disclosed a good value for estimating the risk of recurrence (AUC: 0.849, 95%CI: 0.735-0.963, **Figure 4D**). PBMC LMP7 at discharge was elevated in patients who died (n=3) compared to those who survived (n=159) (*Z*=-2.485, *P*=0.013, **Figure 4E**), with an excellent value to estimate the risk of death (AUC: 0.919, 95%CI: 0.836-1.000, **Figure 4F**).

## 4 Discussion

Though the regulatory role of LMP7 on T cell differentiation is validated by several *in vitro* and *in vivo* studies (11, 12), its correlation with T cell subsets in patients with cerebral diseases is rare. Only one study indicates that LMP7 is positively related to Th1 cells and Th17 cells, but not Th2 cells in Alzheimer’s disease patients (24). This study found that PBMC LMP7 at admission was positively correlated with Th17 cells, but negatively with Th2 cells in AIS patients. The possible explanation could be (1): LMP7 might activate STAT3, and STAT3 activation could promote Th17 cell differentiation (11, 25) (2). LMP7 was negatively correlated with IL-10, and the latter facilitated the differentiation of CD4^+^ T cells into Th2 cells (26, 27). Thus, PBMC LMP7 at admission had a positive correlation with Th17 cells, but a reverse association with Th2 cells in AIS patients. In addition, based on the current literature, it seemed that the inflammation regulatory role of LMP7 was not affected by different types of PBMC subsets (28, 29). On one hand, LMP7 facilitated lymphocyte differentiation to inflammatory effector cells, such as Th17 cells (29). On the other hand, LMP7 inhibited macrophage M2 polarization, promoting inflammation response (28, 30). However, further exploration was warranted for validation.

LMP7 regulates inflammation *in vivo* in many studies (31–33). For example, one study indicates that inhibited LMP7/nuclear factor-kappa B (NF-κB) down-regulates transcription of proinflammatory molecules in mesangial cells in diabetic nephropathy (31). Another study reveals that immunoproteasome-deficient reduces inflammation, proinflammatory and chemotactic cytokines, and IL-17 production in myocarditis mice (33). Nevertheless, the relationship between LMP7 and inflammation levels in AIS patients remains unknown. This study found that PBMC LMP7 at admission was positively associated with CRP in AIS patients. The possible explanation could be that LMP7 promoted inflammation through triggering proinflammatory factors (such as IL-17 and IL-6) (10, 12, 33); therefore, elevated PBMC LMP7 at admission was related to higher level of acute inflammation in AIS patients. Furthermore, this study found that PBMC LMP7 at admission was positively associated with NIHSS score and disease severity (defined by NIHSS score) in AIS patients. The explanation for this may be that according to the results of this study, PBMC LMP7 promoted Th17 cell proportion and inflammation, which could exacerbate neuroinflammation and brain damage (14). Consequently, elevated PBMC LMP7 at admission could reflect exacerbated NIHSS score and disease severity in AIS patients.

More importantly, this study noticed that PBMC LMP7 was decreased at discharge than at admission in AIS patients. The possible explanation could be: Based on the findings of this study, LMP7 was positively related to inflammation and disease severity, meanwhile, the latter would be alleviated after treatment (6). Thus, PBMC LMP7 was decreased from admission to discharge in AIS patients. In addition, this study suggested that PBMC LMP7 at admission possessed limited capability for reflecting risk of disability, recurrence, or death; while PBMC LMP7 at discharge exhibited a weak ability for evaluating disability but a better capacity for estimating recurrence and death in AIS patients. The possible explanation could be (1): PBMC LMP7 accelerated the deterioration of atherosclerosis, leading to a worse prognosis of AIS (34, 35) (2). As revealed by the findings of this study, increased PBMC LMP7 was associated with greater disease severity in AIS patients, and AIS patients with more severe conditions were more likely to experience poor clinical outcomes (36) (3). AIS patients with elevated PBMC LMP7 at discharge were more likely to have severe brain damage, resulting in a worse prognosis (14). Consequently, PBMC LMP7 at discharge was associated with recurrence and death in AIS patients. In addition, according to previous studies, the length of stay ranged from 10 days to 54 days in AIS patients (37–39), with the recurrence rate ranging from 3.3% to 5.9% (8, 39). Thereby, the role of LMP7 at discharge in estimating stroke recurrence and death might be affected by in-hospital recurrence, but this impact was relatively weak. However, it was still necessary to determine LMP7 at discharge of AIS patients due to the fact that the disease condition was fluctuant during hospitalization and some patients after discharge faced the risk of stroke recurrence and death (7, 8).

Some limitations still existed in the study. To begin with, although this study enrolled 162 patients, the relatively low incidence of stroke recurrence and death resulted in data polarity, weakening statistical power for assessing the association of PBMC LMP7 with stroke recurrence and death. In addition, this study evaluated the clinical value of PBMC LMP7 in AIS patients during hospitalization, but its long-term variation and the corresponding prognostic value are unknown.Further, this study did not enroll a control cohort to identify the disease specificity of LMP7 in AIS, which requires future exploration. Finally, the clinical role of PBMC LMP7 in patients with hemorrhagic stroke was unknown.

In summary, elevated PBMC LMP7 at admission reflects reduced Th2 cells, elevated Th17 cells, acute inflammation, and disease severity in AIS patients. Moreover, PBMC LMP7 is decreased from admission to discharge, and its level at discharge is associated with increased risks of disability, stroke recurrence, and death in these patients. Furtherly, it is assumed that LMP7 in lymphocytes and monocytes may promote Th17 cell differentiation and production of inflammatory factors, subsequently aggravating excessive inflammation and cognitive impairment in AIS patients. However, future validation is necessary.

## Data availability statement

The original contributions presented in the study are included in the article/**Supplementary Material**. Further inquiries can be directed to the corresponding author.

## Ethics statement

The studies involving humans were approved by the Ethics Committee of the First Affiliated Hospital of Wenzhou Medical University. The studies were conducted in accordance with the local legislation and institutional requirements. The participants provided their written informed consent to participate in this study.

## Author contributions

LH: Conceptualization, Data curation, Formal Analysis, Investigation, Methodology, Writing – original draft, Writing – review & editing. YZ: Conceptualization, Data curation, Formal Analysis, Investigation, Methodology, Supervision, Writing – original draft, Writing – review & editing.
