# Supplementary material for: Peripheral blood mononuclear cell low molecular mass protein 7 in acute ischemic stroke: vertical change from admission to discharge and correlation with disability, stroke recurrence, and death
Source: Front Immunol. 2024 Feb 8;15:1296835. doi: 10.3389/fimmu.2024.1296835 (PMC10885349; doi:10.3389/fimmu.2024.1296835)
Supplement: Supplementary file 1 [file Table_1.docx]

**Supplementary Table 1.** Association of PBMC LMP7 at admission with Th1 cells, Th2 cells, Th17 cells, CRP, and NIHSS score in AIS patients.

| Characteristics | PBMC LMP7 at admission <1  (n = 80) | PBMC LMP7 at admission ≥1  (n = 82) | *Z/χ^2^* value | *P* value |
| --- | --- | --- | --- | --- |
| Th1 cells (%), median (IQR) | 15.9 (13.3-18.5) | 17.6 (12.7-23.0) | 1.617 ^a^ | 0.106 |
| Th2 cells (%), median (IQR) | 12.4 (9.0-16.7) | 10.6 (8.5-12.3) | 2.503 ^a^ | 0.012 |
| Th17 cells (%), median (IQR) | 2.1 (1.4-3.7) | 3.4 (2.1-5.5) | 3.596 ^a^ | <0.001 |
| CRP (mg/L) |  |  |  |  |
| Median (IQR) | 4.2 (2.5-6.8) | 5.2 (3.3-7.4) | 2.000 ^a^ | 0.045 |
| <5 mg/L, No. (%) | 48 (60.0) | 37 (45.1) | 3.594 ^b^ | 0.058 |
| ≥5 mg/L, No. (%) | 32 (40.0) | 45 (54.9) |  |  |
| NIHSS score |  |  |  |  |
| Median (IQR) | 8.0 (5.0-11.0) | 9.0 (5.0-13.0) | 1.748 ^a^ | 0.081 |
| Mild, No. (%) | 15 (18.8) | 12 (14.6) | 7.584 ^b^ | 0.047 |
| Moderate, No. (%) | 62 (77.5) | 57 (69.5) |  |  |
| Moderate to severe, No. (%) | 3 (3.8) | 8 (9.8) |  |  |
| Severe, No. (%) | 0 (0.0) | 5 (6.1) |  |  |

PBMC, peripheral blood mononuclear cells; LMP7, low molecular mass protein-7; Th1, T helper 1; Th2, T helper 2; Th17, T helper 17; CRP, C-reactive protein; NIHSS, National Institutes of Health Stroke Scale; AIS, acute ischemic stroke; IQR, interquartile range. The median PBMC LMP7 at admission (1.000) was set as a cut-off. The superscript ‘a’ indicated the value was determined by the Wilcoxon rank sum test (*Z* value), and the superscript ‘b’ indicated the value was determined by the *χ^2^* test (*χ^2^* value).
